# Altered Gene Expression in Pulmonary Tissue of Tryptophan Hydroxylase-1 Knockout Mice: Implications for Pulmonary Arterial Hypertension

**DOI:** 10.1371/journal.pone.0017735

**Published:** 2011-03-25

**Authors:** Richard B. Rothman, Jean L. Cadet, Christina M. Dersch, Michael T. McCoy, Elin Lehrmann, Kevin G. Becker, Michael Bader, Natalia Alenina, Michael H. Baumann

**Affiliations:** 1 Translational Pharmacology Section, Intramural Research Program, National Institute on Drug Abuse, National Institutes of Health, Baltimore, Maryland, United States of America; 2 Molecular Neuropsychiatry Research Branch, Intramural Research Program, National Institute on Drug Abuse, National Institutes of Health, Baltimore, Maryland, United States of America; 3 Gene Expression and Genomics Unit, Intramural Research Program, National Institute on Aging, National Institutes of Health, Baltimore, Maryland, United States of America; 4 Max Delbrück Center for Molecular Medicine, Berlin, Germany; Rikagaku Kenkyūsho Brain Science Institute, Japan

## Abstract

The use of fenfluramines can increase the risk of developing pulmonary arterial hypertension (PAH) in humans, but the mechanisms responsible are unresolved. A recent study reported that female mice lacking the gene for tryptophan hydroxylase-1 (*Tph1*(−/−) mice) were protected from PAH caused by chronic dexfenfluramine, suggesting a pivotal role for peripheral serotonin (5-HT) in the disease process. Here we tested two alternative hypotheses which might explain the lack of dexfenfluramine-induced PAH in *Tph1*(−/−) mice. We postulated that: 1) *Tph1*(−/−) mice express lower levels of pulmonary 5-HT transporter (SERT) when compared to wild-type controls, and 2) *Tph1(−/−)* mice display adaptive changes in the expression of non-serotonergic pulmonary genes which are implicated in PAH. SERT was measured using radioligand binding methods, whereas gene expression was measured using microarrays followed by quantitative real time PCR (qRT-PCR). Contrary to our first hypothesis, the number of pulmonary SERT sites was modestly up-regulated in female *Tph1*(−/−) mice. The expression of 51 distinct genes was significantly altered in the lungs of female *Tph1*(−/−) mice. Consistent with our second hypothesis, qRT-PCR confirmed that at least three genes implicated in the pathogenesis of PAH were markedly up-regulated: *Has2*, *Hapln3* and *Retlna*. The finding that female *Tph1*(−/−) mice are protected from dexfenfluramine-induced PAH could be related to compensatory changes in pulmonary gene expression, in addition to reductions in peripheral 5-HT. These observations emphasize the intrinsic limitation of interpreting data from studies conducted in transgenic mice that are not fully characterized.

## Introduction

Fenfluramine and dexfenfluramine (fenfluramines) are anorectic medications that were removed from clinical use. Epidemiological findings have revealed that these drugs increase the risk for two serious cardiovascular side-effects, valvular heart disease (VHD) and pulmonary arterial hypertension (PAH). Most evidence indicates that fenfluramine-induced VHD involves the activation of 5-HT_2B_ receptors localized on heart valves by the metabolite norfenfluramine [1], whereas the mechanisms responsible for PAH remain unresolved.

Based on the fact that fenfluramines are well-established substrates for 5-HT transporter (SERT) proteins, we proposed a “gateway hypothesis” to explain fenfluramine-induced PAH [2]. According to this proposal, SERT proteins localized to pulmonary smooth muscle cells provide gateways for fenfluramines to accumulate inside of cells, where drug molecules then promote mitogenesis via mechanisms that may include direct posttranslational transamination of small proteins [3], [4]. In support of this hypothesis, it is well established that SERT-mediated uptake of 5-HT can induce mitogenic responses in pulmonary smooth muscle cells [5], [6] and similar pathways are activated by fenfluramine itself [7].

An alternative proposal to explain fenfluramine-associated PAH is known as the “5-HT hypothesis” [8]. A key prediction of this hypothesis is that fenfluramine administration increases plasma 5-HT to micromolar (µM) concentrations required to produce vasoconstriction and mitogenesis, which then leads to the development of PAH. Using a novel microdialysis method to measure plasma concentrations of 5-HT in rat whole blood samples, we directly tested the 5-HT hypothesis. Our findings show that acute and chronic administration of fenfluramine can increase plasma concentrations of 5-HT, but the elevations are far below those necessary to produce cardiovascular side-effects [9], [10]. Thus, direct assessment of plasma 5-HT suggests that the 5-HT hypothesis cannot explain the pathology of fenfluramine-induced PAH.

Recently, investigators have used knockout mice which lack the gene for tryptophan hydroxylase-1 (*Tph1*(−/−) mice) to examine the role of 5-HT in mediating dexfenfluramine-induced PAH [11]. Tryptophan hydroxylase-1 is the chief enzyme responsible for synthesis of peripheral 5-HT [12], and *Tph1*(−/−) mice display markedly reduced concentrations of 5-HT in whole blood (92% decrease), platelets (90% decrease) and plasma (80% decrease) [13], [14], [15]. Dempsie et al. [11] reported that chronic oral administration of dexfenfluramine (5 mg/kg/day) for 28 days produces PAH in wild-type (WT) female mice but not in *Tph1*(−/−) female mice. Curiously, dexfenfluramine does not produce PAH in WT male mice (Dr. M. MacLean, personal communication), demonstrating the sex-specific nature of the observed pathology. In view of the substantial reductions in peripheral 5-HT present in *Tph1*(−/−) mice, the authors concluded that dexfenfluramine fails to produce PAH due to lack of peripheral 5-HT.

There are caveats to interpreting the effects of dexfenfluramine in *Tph1*(−/−) mice. First, Dempsie et al. [11] never actually measured plasma 5-HT in WT and *Tph1*(−/−) mice. There is an assumption that dexfenfluramine would not increase plasma 5-HT in *Tph1*(−/−) mice, but data from rats suggest otherwise. Zolkowska et al. [10] showed that chronic fluoxetine administration (10 mg/kg/day) for 14 days decreased whole blood 5-HT by nearly 80%, but acute fenfluramine still caused modest increases in plasma 5-HT. Second, it is well known that a single gene deletion can lead to adaptive changes in the expression of many other genes. For instance, deletion of the gene for vasoactive intestinal peptide (VIP) in mice causes changes in the expression of many pulmonary genes, including those thought to contribute to the PAH phenotype [16]. Transgenic mice that express a truncated form of bone morphogenetic protein receptor type II (BMPR2) demonstrate altered expression of 181 unique named genes [17]. It seems possible that changes in the expression of genes unrelated to 5-HT synthesis may contribute to the protection of female *Tph1*(−/−) mice from dexfenfluramine-induced PAH.

In view of the above-mentioned considerations, we tested two hypotheses related to the lack of PAH in *Tph1*(−/−) mice. First, we hypothesized that *Tph1*(−/−) mice express lower levels of SERT than WT controls. A decrease in SERT expression would be predicted to impede the entry of dexfenfluramine into cells, thereby reducing the propensity for developing PAH. Second, we hypothesized that *Tph1(−/−)* mice display compensatory changes in the expression of genes unrelated to 5-HT synthesis that provide protection from PAH. Our findings demonstrate that genetic deletion of tryptophan hydroxylase-1 produces a modest up-regulation of pulmonary SERT binding sites (i.e., 30–40% elevation) and an increase in the expression level of several genes implicated in the pathogenesis of PAH. Thus, the resistance of female *Tph1(−/−)* mice to dexfenfluramine-induced PAH could be related to mechanisms that do not involve decreased peripheral 5-HT.

## Results

### SERT binding experiments

As shown in Table 1, the binding density (B_MAX_) for pulmonary SERT was increased by 30–40% in both male and female *Tph1*(−/−) mice as compared to WT controls. In brain tissue, SERT B_MAX_ for *Tph1*(−/−) and WT mice was not significantly different (Table 2). The binding experiments revealed important sex differences in the density of SERT proteins. The pulmonary SERT B_MAX_ was significantly higher in males than in females, whereas the brain SERT B_MAX_ was lower in males than in females.

**Table 1:** SERT Binding Parameters in Mouse Lung.

	B_MAX_(fmol/mg protein±SD)	K_D_(nM±SD)
Females		
*Tph1*(+/+) (WT) (n = 4)	95±9	0.97±0.07
*Tph1*(−/−) (n = 5)	131±13*	0.76±0.08
Males		
*Tph1*(+/+) (WT) (n = 11)	145±9#	0.71±0.02
*Tph1*(−/−) (n = 7)	186±12* †	0.87±0.05*

[^125^I]RTI-55 binding assays were conducted as described in METHODS.

*p<0.05 compared to the corresponding WT mouse of the same sex;

# p<0.05 when compared to the WT female mouse;

^†^p<0.05 when compared to the *Tph1*(−/−) female mouse (Student's t-test, p<0.05).

**Table 2:** SERT Binding Parameters in Mouse Brain.

	B_MAX_(fmol/mg protein±SD)	K_D_(nM±SD)
Females		
*Tph1*(+/+)(WT) (n = 5)	659±19	0.51±0.01
*Tph1*(−/−) (n = 5)	692±26	0.50±0.01
Males		
*Tph1*(+/+) (WT)(n = 5)	589±25*	0.51±0.01
*Tph1*(−/−) (n = 5)	618±30*	0.51±0.01

[^125^I]RTI-55 binding assays were conducted as described in METHODS.

*p<0.05 compared to the corresponding females (Student's t-test).

### Microarray findings

The findings summarized in Table 3 show that more than 20 genes were significantly up-regulated in *Tph1*(−/−) mice when compared to WT. The most markedly up-regulated gene was hemoglobin, beta adult minor chain (*Hbb-b1*). Other up-regulated genes included hyaluronan and proteoglycan link protein 3 (*Hapln3*), dihydropyrimidine dehydrogenase (*Dpyd*), keratin 17 (*Krt-17*), solute carrier family 17 (sodium-dependent inorganic phosphate co-transporter) (*Slc17a8*), hyaluronan synthase 2 (*Has2*), and resistin like alpha (*Retnla*), among others. Table 4 shows the list of 23 down-regulated genes which included G protein receptor 141 (*Grp141*), DNAJ (HSP40) homolog, subfamily 3 (*DNAjb3*), and solute carrier family 6 (neurotransmitter transporter, L-proline), member 7 (*Slc6a7*).

**Table 3:** Microarray Results: Up-regulated Genes.

*Tph1*/(−/−)WTFold-Change	Gene Symbol	Common Name	Description
67.18	*Hbb-b2*	Hbb-b1	hemoglobin, beta adult minor chain
24.57	*Hapln3*	Hapln3	hyaluronan and proteoglycan link protein 3
14.63	*Dpyd*	Dpyd	dihydropyrimidine dehydrogenase
14.25	*Etv4*	Etv4	ets variant gene 4, E1AF
13.17	*Derl3*	Derl3	Der1-like domain family, member 3
9.70	*Fuca1*	Fuca1	fucosidase, alpha-L- 1, tissue
9.68	*Krt17*	Krt1-17	keratin 17
9.36	*Slc17a8*	Slc17a8	solute carrier family 17, member 8
9.31	*Rg9mtd3*	Rg9mtd3	RNA (guanine-9-) methyltransferase domain containing 3
9.05	*Olig1*	Olig1	oligodendrocyte transcription factor 1
8.71	*Ybx2*	Ybx2	Y box protein 2
8.21	*Il5*	Il5	interleukin 5
8.00	*Has2*	Has2	hyaluronan synthase 2
7.86	*Syt13*	Syt13	synaptotagmin XIII
7.58	*Lrrc9*	Rik	leucine-rich repeat-containing 9
7.37	*Stra6*	Stra6	stimulated by retinoic acid gene 6
7.11	*Crygc*	Crygb	crystallin, gamma C
2.97	*Car11*	Car11	carbonic anhydrase 11
2.86	*Bcat2*	Bcat2	branched chain aminotransferase 2, mitochondrial
2.59	*Serpinh1*	Serpinh1	serine (or cysteine) peptidase inhibitor, clade H, member 1
2.29	*Retnla*	Retnla	resistin like alpha
2.22	*Gldc*	Gldc	glycine decarboxylase
2.01	*Luzp2*	Luzp2	leucine zipper protein 2

These data were generated from lung tissue harvested from WT and *Tph1*(−/−) female mice (n = 6 per group). The above changes were statistically significant (p<0.025).

**Table 4:** Microarray Results: Down-regulated Genes.

*Tph1*(−/−)/WTFold-Change	Gene Symbol	Common Name	Description
−2.00	*Dlc1*	HP; Arhgap7	deleted in liver cancer 1
−2.01	*Slc6a7*	Slc6a7	solute carrier family 6, member 7
−2.46	*Tmco7*	AW413431	transmembrane and coiled-coil domains 7
−4.35	*Psmd8*	Psmd8	proteasome 26S subunit, non-ATPase, 8
−5.43	*V1rh13*	V1rh13	vomeronasal 1 receptor, H13
−6.49	*Gpr141*	Gpr141	G protein-coupled receptor 141
−7.09	*Pdzd3*	Pdzk2	PDZ domain containing 3
−9.52	*Foxd4*	Foxd4	forkhead box D4
−13.87	*Crabp1*	Crabp1	cellular retinoic acid binding protein I
−14.29	*Slc2a9*	Slc2a9	solute carrier family 2 (glucose transporter), member 9
−14.71	*Dnajb3*	Dnajb3	DnaJ (Hsp40) homolog, subfamily B, member 3
−21.28	*Picalm*	Picalm	phosphatidylinositol binding clathrin assembly protein
−44.44	*AI451617*	LOC209387	expressed sequence AI451617
−111.98	*Trim30*	Trim30	tripartite-motif protein 30

These data were generated from lung tissue harvested from WT and *Tph1*(−/−) female mice (n = 6 per group). The above changes were statistically significant (p<0.025).

### Quantitative real-time PCR results

**Figure 1:**
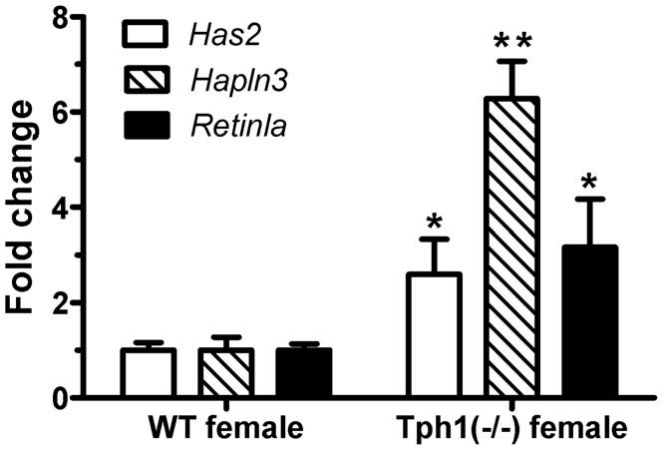
Quantitative real-time PCR was conducted as described in MATERIALS AND METHODS with pulmonary tissue harvested from WT and *Tph1* (−/−) female mice (n = 6). *p<0.05, **p<0.001 when compared to WT (ANOVA).

We used quantitative real-time PCR to confirm the changes in expression of three genes that have been implicated in the pathogenesis of PAH: *Has2*, *Hapln3* and *Retnla*. The first two genes relate directly to hyaluronic acid, a component of the extracellular matrix that has been implicated in PAH [18]. *Retnla* (resistin like alpha) has also been strongly linked to PAH [19]. Consistent with the array results, all three genes were significantly up-regulated in the *Tph1*(−/−) mice in comparison the WT control mice as depicted in Fig. 1.

## Discussion

Epidemiological evidence links the use of aminorex, fenfluramine and dexfenfluramine to the development of PAH in human subjects [for review see [20]]. It is noteworthy that aminorex poses a much greater risk for PAH when compared to fenfluramines, but the underpinnings of pulmonary pathology remain unclear in both cases. Efforts to study the mechanisms involved with anorexigen-induced PAH have been stymied by the failure of these drugs to reliably produce pulmonary pathology in laboratory animal species. As reviewed by Weir [20], chronic administration of aminorex does not produce PAH in monkeys, calves, rats or dogs. Similarly, chronic administration of fenfluramines does not produce PAH in male rats [21], [22] or mice [23]. Given this information, it is a remarkable finding that chronic administration of dexfenfluramine produces PAH in female mice of the C57BL/6 strain [11]. Because female mice of this strain appear to be unique in their response to chronic dexfenfluramine, this mouse model could be useful for dissecting out the pathogenesis of anorexigen-induced PAH. Indeed, it would be interesting for future studies to compare overall gene expression patterns of male versus female C57BL/6 mice to identify factors responsible for the sex-specific propensity of females to develop PAH.

Dempsie et al. [11] reported that deletion of the gene for tryptophan hydroxylase-1 in female C57BL/6 mice provided protection from PAH produced by chronic dexfenfluramine. Because *Tph1*(−/−) mice are known to have markedly lower levels of peripheral 5-HT, a logical conclusion is that plasma 5-HT is necessary for the development of dexfenfluramine-induced PAH in these mice. As noted in the Introduction, two assumptions underlie this conclusion. The first assumption is that dexfenfluramine does not increase plasma 5-HT in *Tph1*(−/−) mice, a possibility that has not been examined. Data from rats show that fenfluramine can increase plasma 5-HT even under conditions where platelet 5-HT is markedly reduced [10]. The second assumption was that the only change of significance in *Tph1*(−/−) mice was deletion of the gene for tryptophan hydroxylase-1. It is well documented that transgenic mice can have compensatory changes in gene expression, so it seems plausible that alterations in the expression of genes unrelated to 5-HT synthesis could contribute to the failure of dexfenfluramine to produce PAH in female *Tph1*(−/−) mice.

The first hypothesis tested in the present study was that pulmonary SERT might be down-regulated in *Tph1*(−/−) mice. Contrary to this proposal, SERT was modestly up-regulated in pulmonary membranes prepared from both male and female *Tph1(−/−)* mice. One simple interpretation of the SERT data is that *Tph1*(−/−) mice display increased SERT B_MAX_ in lung tissue to maintain optimal levels of intracellular 5-HT in response to markedly reduced plasma 5-HT. Studies have demonstrated that SERT is expressed almost exclusively on pulmonary arteries in rat lung (Morecroft et al. 2005), but the precise localization of SERT in mouse lung has not been determined. Surprisingly, we found that male C57BL/6 WT mice had a 50% higher SERT B_MAX_ than female WT mice of this strain, so it seems that elevated SERT B_MAX_ does not predispose male mice to the development of dexfenfluramine-induced PAH. Other studies have shown that transgenic mice over-expressing SERT (SERT+ mice) have increased right ventricular pressure and develop PAH more robustly in response to hypoxia than WT controls [11], [24], [25]. To our knowledge, the possibility that the expression of other genes related to the pathogenesis of PAH are altered in SERT+ mice has not been examined.

A major finding of the present study is that expression levels of numerous genes unrelated to 5-HT are altered in female *Tph1*(−/−) mice. The data in Tables 3 and 4 demonstrate that 28 pulmonary genes were significantly up-regulated while 23 genes were down-regulated in *Tph1*(−/−) mice. Many of these changes in gene expression exceeded 10-fold relative to WT controls. The physiological relevance of these changes is not immediately clear, but expression levels of specific genes related to the pathogenesis of PAH were significantly altered. For example, it is well known that PAH is accompanied by the turnover of extracellular matrix constituents, including hyaluronic acid [18]. Therefore, it is notable that *Tph1*(−/−) mice over-expressed genes related to hyaluronic acid synthesis and deposition: *Has2* and *Hapln3*. Moreover, *Retnla* (resistin like alpha, HIMF, FIZZ1) was also up-regulated in *Tph1*(−/−) mice, and this gene has been implicated in the development of hypoxia-induced PAH [19], [26]. As reviewed by Fan et al. [19], *in vivo* knockdown of *Retnla* had a protective effect on hypoxia-induced PAH, whereas over-expression of *Retnla* in the lung caused PAH. Direct extrapolation of our data predicts that *Tph1*(−/−) mice would be more prone to develop PAH in response to hypoxia than WT mice. The relevance of this prediction to dexfenfuramine-induced PAH is not clear, especially in light of the extensive changes in gene expression observed in these mice. Viewed collectively, our data support the hypothesis that changes in the expression of genes unrelated to 5-HT synthesis may contribute to the absence of dexfenfluramine-induced PAH in female *Tph1*(−/−) mice. It must be noted that since whole lungs were used in our experiments, we cannot determine which specific tissue type (vascular, endothelial, pulmonary, etc.) exhibits the changes in gene expression noted here.

In summary, the expression of numerous pulmonary genes are altered in *Tph1*(−/−) mice, including some genes that have been implicated in PAH. The physiological significance of these changes in gene expression remains to be determined. Based on our data, it seems reasonable to conclude that the insensitivity of female *Tph1*(−/−) mice to dexfenfluramine-induced PAH could be related to compensatory changes in non-serotonergic gene expression, in addition to lower levels of peripheral 5-HT. These observations emphasize the intrinsic limitation of interpreting data from studies using transgenic mice without first performing thorough phenotypic characterization.

## Materials and Methods

### Animals and tissue collection

Mice were maintained in IVC cages (Tecniplast Deutschland, Hohenpeissenberg, Germany) under standardized conditions with an artificial 12-h dark–light cycle, with free access to standard chow (SSNIFF Spezialitäten, Soest, Germany) and drinking water ad libitum. Local German authorities approved the studies with standards corresponding to those prescribed by the American Physiological Society. Male and female *Tph1*(−/−) (7 generation backcross to C57BL/6 background) mice and corresponding C57BL/6 controls (3.5 to 5 month old) were used for the experiments. To collect the organs, animals were anesthetized by intraperitoneal (i.p.) ketamine (100 mg/kg) and xylazine (50 mg/kg) and perfused with PBS to wash out blood. Lungs and brains were isolated and immediately snap-frozen in liquid nitrogen. Mice were not treated with any pharmacological agents, except for anesthetics at the time of perfusion. The animal work was conducted according to relevant national and international guidelines and was approved by the local authorities (Landesamt für Gesundheit und Soziales, Berlin; approval ID: T0042/06). Tissues were harvested in the laboratory of Dr. Bader and shipped to the laboratory of Dr. Rothman. The lungs were not examined in terms of pathology and appearance because no abnormalities were noted in previous studies using these mice.

### Membrane preparation for bindings assays

Whole lungs and brains were frozen at −80°°C until the day of assay. On the day of the assay, frozen lungs or brains were prepared as described [27] with minor modifications. The frozen tissue was homogenized with a polytron (setting 6, 15 sec) using 10 ml of tissue buffer (TB) per sample. The TB was 50 mM Tris-HCl, 150 mM NaCl, 1 mM EDTA, 10 mM MgCl_2_, with 10% glycerol, 500 µg/ml soybean trypsin inhibitor, 10 mM benzamidine, 1 µg/ml each of leupeptin, bacitracin, pepstatin A, and antipain, pH 7.4. Homogenized lung tissue was centrifuged at 1200× g for 5 min at 4°C. The resulting supernatant was centrifuged at 48,000× g for 30 min at 4°C; the pellet was resuspended and centrifuged at 48,000× g for 30 min at 4°C. The pellet was resuspended in ice cold binding buffer (BB: 55.2 mM sodium phosphate pH 7.4) and used in the assay. Homogenized brain tissue was centrifuged at 30,000× g for 10 min at 4°C. The pellet was resuspended and centrifuged at 30,000× g for 10 min at 4°C after which the pellet was resuspended in BB and used in the assay. Typical protein values, as determined by the Lowry method, were 0.05–0.07 mg/ml.

### Radioligand binding procedures

SERT sites were labeled using [^125^I]RTI-55 (PerkinElmer Life and Analytical Sciences, SA = 2200 Ci/mmol). The binding assays proceeded as follows. Polystyrene test tubes (12×75 mm) were prefilled with 100 µl of drug (RTI-55), 100 µl of radioligand (final concentration ∼0.01 nM) and 50 µl of a “blocker.” The blocker was used at a final concentration of 100 nM GBR12935 and 100 nM desipramine to prevent [^125^I]RTI-55 from binding to dopamine or norepinephrine transporters. RTI-55 and the blocker were made up in BB containing 1 mg/ml BSA (BB/BSA). The radioligand was made up in a protease inhibitor cocktail containing BB/BSA (chymostatin [25 µg/ml], leupeptin [25 µg/ml], EDTA [1 mM], EGTA [1 mM]). The assay was initiated by the addition of 750 µL of the prepared membranes. Following an 18–20 hr incubation at 4°C, samples were filtered over Whatman GF/B filters which were presoaked in wash buffer (ice-cold 10 mM Tris-HCl, pH 7.4 containing 150 mM NaCl) containing 2% polyethylenimine. Nonspecific binding was determined using 1 µM indatraline.

### Binding assay design, analysis, and statistics

Ten point inhibition curves were generated by displacing [^125^I]RTI-55 binding using unlabeled RTI-55 (0.02–10 nM [brain] or 0.02–82 nM [lung]). The data of several experiments were pooled and fit to the one site binding model for the best-fit estimates of the B_MAX_ and KD using MLAB-PC as previously described [28]. Statistical significance (p<0.05) between binding parameters was determined by the Student's t-test.

### RNA extraction

Whole lung tissue was powdered under liquid nitrogen in a mortar and pestle. Total RNA was isolated from 30 mg of powdered tissue using Qiagen RNeasy Mini kit (Qiagen, Valencia, CA) according to the manufacturer's instructions. RNA integrity was assessed using an Agilent 2100 Bioanalyzer (Agilent, Palo Alto, CA) and showed no degradation.

### Microarray hybridization, scanning, and data analysis

Microarray hybridization was carried out using Illumina's Mouse-ref 8 Expression BeadChips arrays (22,523 probes) (Illumina Inc., San Diego, CA). In brief, a 600 ng aliquot of total RNA from each pulmonary sample obtained from female C57BL/6 WT and female *Tph1*(−/−) mice (n = 6) was amplified using Ambion's Illumina RNA Amplification kit (Ambion, Austin, TX). Single-stranded RNA (cRNA) was generated and labeled by incorporating biotin-16-UTP (Roche Diagnostics, Indianapolis, IN). Seven hundred fifty ng of each cRNA sample was hybridized to Illumina arrays at 55°C overnight according to the Illumina Whole-Genome Gene Expression Protocol for BeadStation (Illumina Inc.). Hybridized biotinylated cRNA was detected with Cyanine3-streptavidin (Amersham Biosciences, Piscataway, NJ) and quantified using Illumina's BeadStation 500GX Genetic Analysis Systems scanner. The microarray data reported in the manuscript are in accordance with MIAME guidelines. The Illumina BeadStudio software was used to measure fluorescent hybridization signals. Data were extracted by BeadStudio (Illumina). The array data, generated with a total of 6 mice per group, were processed by GenomeStudio version 1.6.0. Data were then imported and analyzed using GeneSpring software v. 7 (Silicon Genetics, Redwood City, CA, USA). A gene was identified as changed if it showed increases or decreases in expression according to an arbitrary cut-off of 2-fold changes at p<0.025.

### Quantitative Real-time PCR

Individual total RNA obtained from 6–8 mice per group was reverse-transcribed with oligo dT primers and RT for PCR kit (Clontech, Mountain View, CA). PCR experiments were done using the LightCycler 480 II (Roche, Indianapolis, IN) and iQ SYBR Green Supermix (BioRad, Hercules, CA) according to the manufacturer's protocol. Sequences for gene-specific primers corresponding to PCR targets were obtained using LightCycler Probe Design software (Roche). The primers were synthesized and HPLC-purified at the Synthesis and Sequencing Facility of Johns Hopkins University (Baltimore, MD). Quantitative PCR values were normalized using OAZ1 (a gene for ornithine decarboxylase antizyme 1) based on the paper by De Jong et al [29].

### Statistical analysis of PCR data

Statistical analysis for the PCR data was performed using analysis of variance (ANOVA) followed by post-hoc analyses (StatView 4.02, SAS Institute, Cary, NC). Values are shown as means ± SEM. The null hypothesis was rejected at p<0.05.
